# Supplementary material for: Low level visual features support robust material perception in the judgement of metallicity
Source: Sci Rep. 2021 Aug 12;11:16396. doi: 10.1038/s41598-021-95416-6 (PMC8361131; doi:10.1038/s41598-021-95416-6)
Supplement: Supplementary file 1 — Supplementary Information. [file 41598_2021_95416_MOESM1_ESM.pdf]

# Supplementary Information

## Low Level Visual Features Support Robust Material Perception in the Judgement of Metallicity

**Joshua S. Harvey**[1,2,3*] **and Hannah E. Smithson**[2]

[1]NYU Langone Health, Neuroscience Institute, New York, NY 10016, USA
[2]Oxford University, Department of Engineering Science, Oxford, OX1 3PJ, UK
[3]Oxford University, Department of Experimental Psychology, Oxford, OX2 6GG, UK
[*]joshua.harvey@nyulangone.org

## Supplementary Movie 1

Decreasing metal smoothness, keeping coating bumpiness at minimum.

## Supplementary Movie 2

Increasing coating bumpiness, keeping metal smoothness at maximum.

## Supplementary Movie 3

Increasing viewing angle.

## 1 The parameters of the stimuli

In this study our stimuli were physically-based renders of metal objects of varying surface smoothness with bumpy, transparent coatings. By varying both the smoothness of the metal and the level of bumpiness of the coating, we are able produce a wide range of images with varying surface appearances. Here, we explain in greater detail what each dimensions of the stimulus set does, and how it effects image properties.

### 1.1 Metal smoothness

Surface smoothness, the inverse if surface roughness, is defined as the width of the distribution of microfacet angles on the surface of the mesh. The microfacet distribution of a perfectly smooth surface has no variance, all microfacets have the same angle as the surface, and for a metal, all incoming light is scattered symmetrically from the normal of the surface plane. For rougher metal surfaces, the specular reflection spreads as the variance of the distribution of microfacet angles increases. This is similar to convolving the reflected image with a gaussian, blurring it. However, due to the macroscale shape of the object, the effect is slightly different to a smooth metal object in a blurred environment (i.e. filtering out some of the higher frequency spherical harmonics of the environment) or simply blurring the image of a smooth metal object in the environment.

### 1.2 Coating bumpiness

For the second dimensions of the stimulus set, we vary the bumpiness of a transparent coating on the metal object. This is defined as a single value, the strength of a *Displace* modifier on the coating mesh. This modifier applies a displacement texture to the mesh, elevating or depressing it along the mesh normal. The texture used is the *Stucci* noise pattern, a random displacement field that simulates a Stucco wall or similar decorative process.

One question is why a coating is necessary, rather than applying bumpiness directly to the metal surface of the object? Doing so gives an appearance like hammered silver. While a plane piece of smooth metal mirrors the environment (Fig. S1a), adding bumpiness scrambles the organisation of this image, affecting both its spatial and chromatic statistics, and introducing discontinuities (Fig. S1b). Adding the same level of bumpiness to a transparent coating sitting above the metal surface alters the reflection of the environment in a smoother, subtler way, analogous to a local disarray vector field (Fig. S1c), preserving much of the spatial and chromatic statistics of the environment.

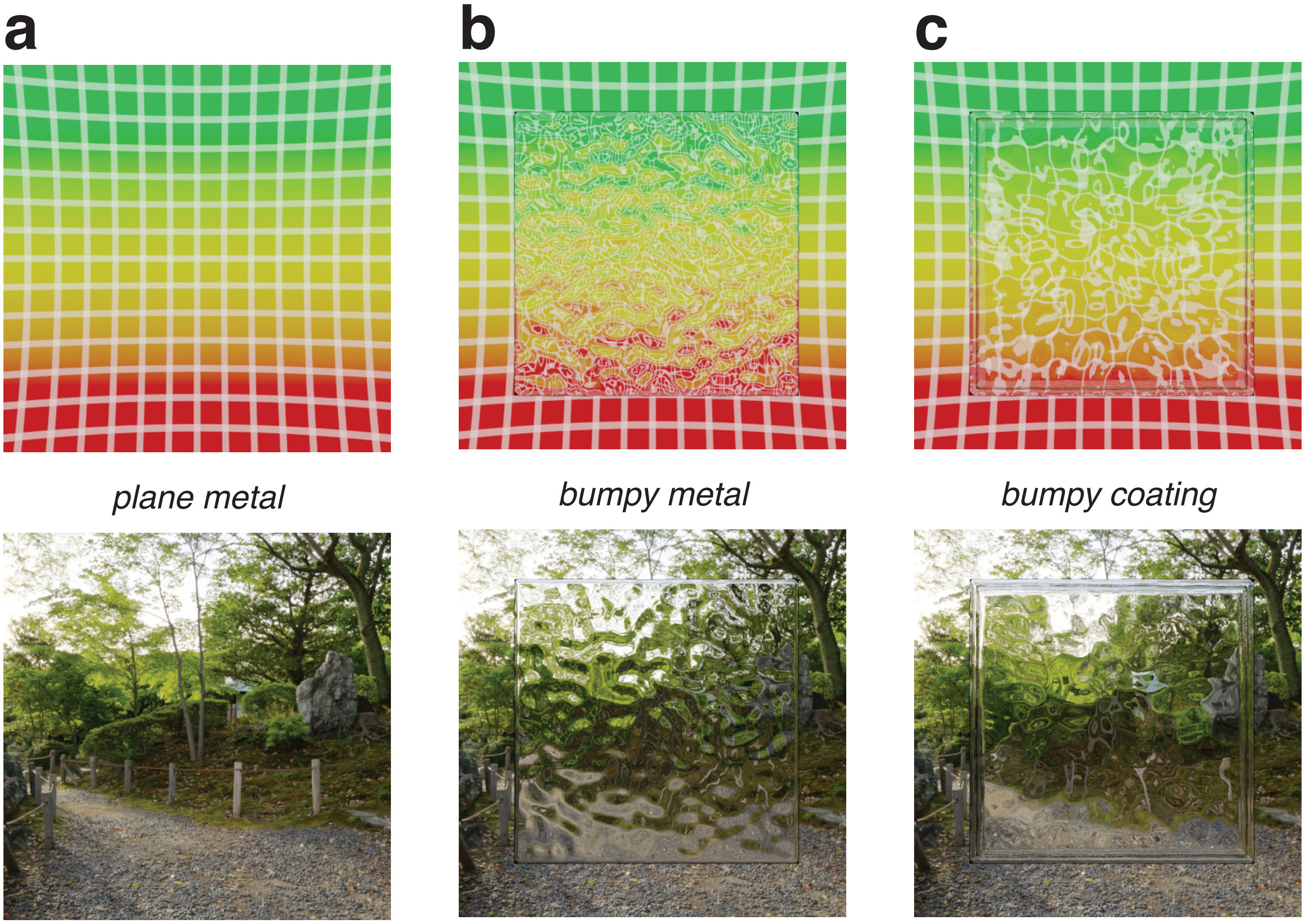


**Figure S1.** The effect that bumpiness has when applied to a metal surface or a transparent coating. (**a**) A plane sheet of smooth metal reflects the environment. Upper panel: an artificial environment with a hue gradient passing through yellow on the horizon, and a regular white grid. Lower panel: a light probe of the Ninomaru Teien garden taken by Greg Zaal. (**b**) Deforming the metal sheet with bumpiness scrambles the spatial and chromatic statistics of the reflected environment, and introduces discontinuities. This is clear in the artificial environment; the reflected image is no longer a uniform gradient from green at the top to red at the bottom, and the grid has been warped and compressed beyond recognition. (**c**) A transparent coating with the same geometry as in **b** (only the front of the mesh is bumpy) modulates the reflected image in a more subtle, continuous manner; the hue gradient is retained, and the grid is of the same scale, albeit subject to a local disarray field.

## 2 The performance of global contrast metrics in estimating metallicity

To see whether the performance of observers could be replicated by global contrast metrics, we tested the performance of models that took the output of such metrics in the conjoint measurement task. The results are shown in Figure S2.

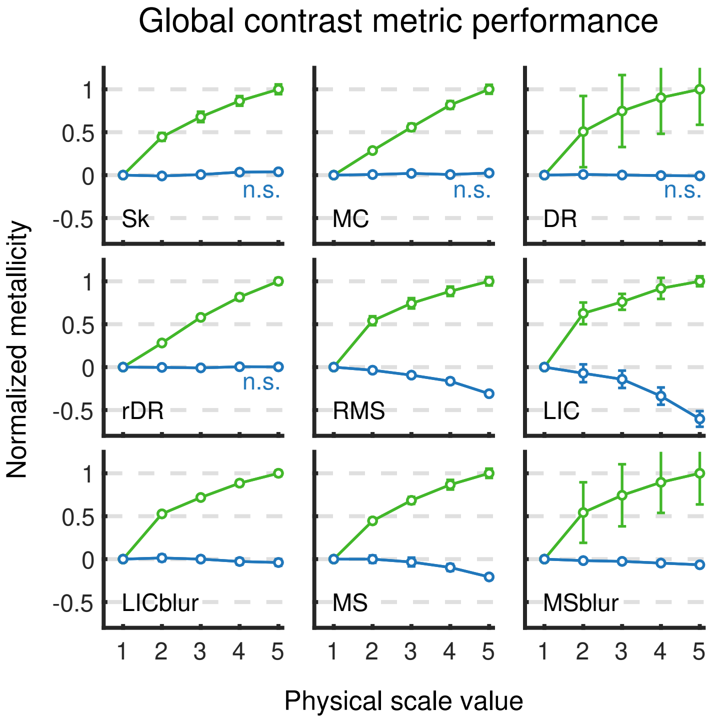


**Figure S2.** The conjoint measurement performance of models estimating metallicity according to different global contrast metrics, as a function of increasing metal smoothness (green) and coating bumpiness (blue). Some metrics give rise to independent conjoint measurement models, where coating bumpiness has no significant effect on metallicity judgment (indicated as 'n.s.' on the plot).

## 3 Steerable pyramid synthesis for changes in viewing angle

To see whether steerable pyramid coefficients provided a robust, causal determination of material appearance, regardless of viewing angle, we synthesised images by transferring pyramids across both parameter levels (metal smoothness and coating bumpiness) and viewing angle with the results shown in Figure S3.

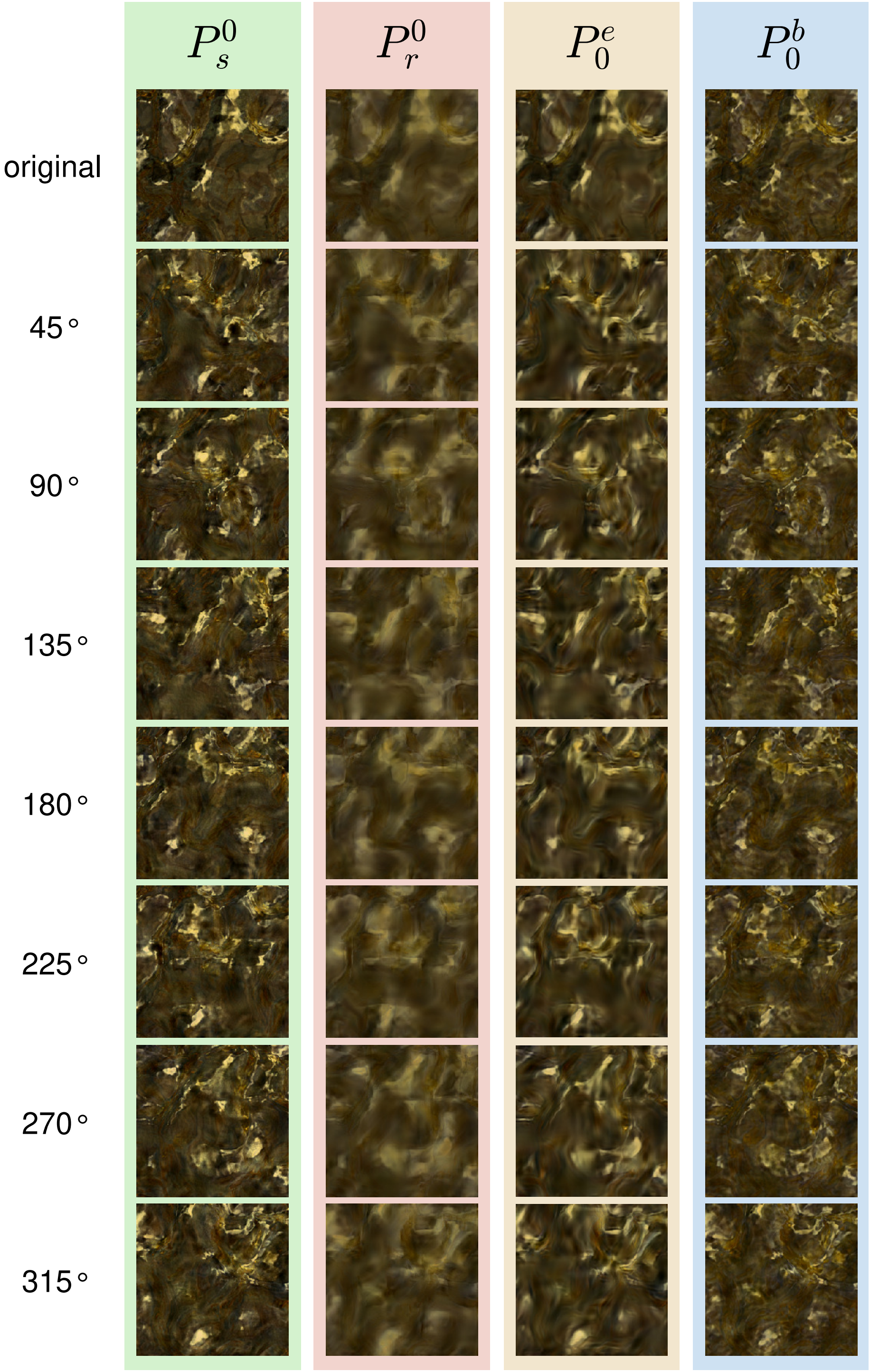


**Figure S3.** Steerable pyramid swaps across viewing angles. For each image, the object from the center of the parameter space at a viewing angle specified in the left column is used as the base image for pyramid synthesis. The pyramid transferred is either for the smoothest ($P_s^0$) or roughest ($P_r^0$) metal, or the most even ($P_0^e$) or bumpy coating ($P_0^b$).

The synthesis is most successful when pyramids are transferred across objects of the same viewing angle (top row of images). Performance is still reasonably good when transferring across different viewing angles, although some of the fine details in the images show minor issues. This suggests that although the steerable pyramid captures much of the appearance properties of our stimuli, it does not perfectly account for the interactions between macro-scale shape and illumination.
